# A Case Report on Trastuzumab Emtansine (T-DM1) in a Patient With Human Epidermal Growth Factor Receptor 2 (HER2)-Positive Metastatic Breast Cancer and Brain Metastases: Long-Term Treatment and Survival

**DOI:** 10.7759/cureus.63589

**Published:** 2024-07-01

**Authors:** Vipul Doshi, Vipulkumar Thummar, Priya Mehta

**Affiliations:** 1 Medical Oncology, Solapur Cancer Centre, Solapur, IND; 2 Medical Affairs, Zydus Lifesciences Ltd, Ahmedabad, IND

**Keywords:** brain metastases, metastasis, breast cancer, her2-positive, biosimilar, t-dm1

## Abstract

Breast cancer remains the most common cancer in women worldwide. Among women with breast cancer, brain metastases are very prevalent among HER2-positive and affect those in the advanced stages of the disease. Various factors, including molecular subtypes, performance status, extracranial disease status, leptomeningeal metastasis, and the number of lesions, significantly influence the prognosis of patients with brain metastases from breast cancer (BCBrM). Understanding and addressing the specific risks associated with different breast cancer subtypes is crucial for developing tailored and effective medical treatments. This report presents a case of a breast cancer patient with recurrent disease and brain metastases who achieved long-term survival following a treatment regimen that included radiotherapy and a T-DM1 biosimilar.

## Introduction

Breast cancer stands as the most prevalent cancer in women, and it emerges as the second most frequent cause of brain metastases (BrM). Various reports suggest an incidence ranging from 10% to 30% in breast cancer patients. The likelihood of developing BrM is contingent upon the subtype, with a heightened occurrence in individuals with human epidermal growth factor receptor 2 (HER2)-positive and triple-negative breast cancer [1].

Within the HER2-positive breast cancer subtype, the occurrence of BrM diagnosis is notable, affecting 25% to 50% of women in advanced stages of the disease [1]. This underscores the importance of understanding and addressing the specific risks associated with different breast cancer subtypes, contributing to more tailored and effective medical approaches.

Molecular subtypes, performance status, extracranial disease status, leptomeningeal metastasis, and the number of lesions all emerge as independent factors influencing the prognosis of individuals with brain metastases from breast cancer (BCBrM) [2].

Notably, advancements in systemic therapy options have led to an improved prognosis for patients with HER2-positive breast cancers compared to those with HER2-negative BCBMs. In patients with good performance status, the median survival surpasses 20 months [3].

As of now, trastuzumab emtansine (T-DM1) stands as the standard therapy for HER2-positive breast cancer patients experiencing recurrence or disease progression after receiving treatment with trastuzumab and pertuzumab [1].

Herein, we present a case of a breast cancer patient with recurrent disease and brain metastases who achieved long-term survival following a treatment regimen comprising radiotherapy and T-DM1 biosimilar. This underscores the potential efficacy of this therapeutic approach in extending survival outcomes for individuals facing similar clinical scenarios.

## Case presentation

The case involves a 53-year-old female with a documented history of left breast cancer. The initial diagnosis was established via fine needle aspiration cytology (FNAC) in April 2018, revealing dysplastic cells indicative of ductal carcinoma. A subsequent PET-CT scan identified a fluorodeoxyglucose (FDG)-avid lesion in the outer quadrant of the left breast with involvement of regional lymph nodes, though no distant organ metastasis was detected.

A left modified radical mastectomy (MRM) was conducted in April 2018. Histopathological examination confirmed a diagnosis of invasive ductal carcinoma (IDC), grade III. The tumor was characterized by the absence of Estrogen and Progesterone receptors (ER- and PR-negative), but was positive for HER2. The surgical margins were clear; however, axillary clearance indicated metastasis in 20 out of 32 lymph nodes.

The patient underwent adjuvant chemotherapy, receiving four cycles of adriamycin and cyclophosphamide, followed by 12 cycles of weekly paclitaxel and trastuzumab from June 2018 to August 2019. Maintenance therapy with Trastuzumab was continued for one year. Additionally, the patient received radiation therapy in January 2019.

In June 2020, a whole-body PET-CT scan revealed the resolution of the previously identified breast lesion and regional lymph node involvement. However, a new FDG-avid lesion was detected in the left frontal lobe of the brain, suggesting metastasis. An MRI scan of the brain confirmed the presence of a lobulated, intensely enhancing lesion in the left high frontal region, accompanied by perilesional edema. This lesion was compressing the left lateral ventricle and causing a midline shift.

In July 2020, excisional biopsy and craniotomy were performed to remove the frontal brain mass, which was identified as a metastatic poorly differentiated adenocarcinoma consistent with the breast primary. Subsequent post-operative brain imaging revealed no residual lesion or recurrence. Whole-brain palliative radiation therapy was given in August 2020.

By October 2020, no evidence of residual brain lesions was observed. However, in January 2021, a PET CT indicated the resolution of the FDG mass in the left frontal lobe, but new FDG avid deposits were discovered in both lobes of the liver. The patient was started on oral chemotherapy with lapatinib and capecitabine thereafter. Imaging studies in subsequent months showed variable findings, including post-operative changes in the left frontal bone, gliosis in the left frontal region, and chronic ischemic changes in bilateral periventricular and subcortical white matter.

**Figure 1:**
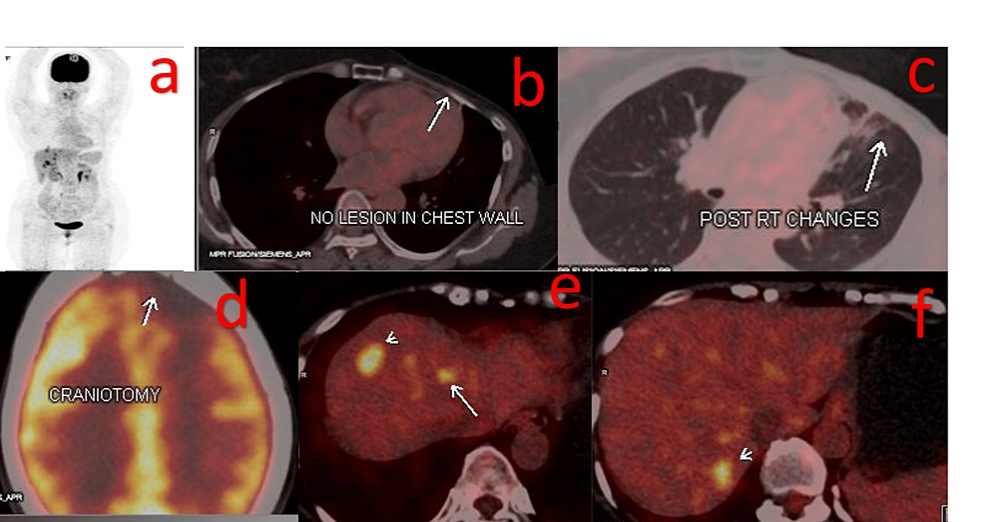
PET-CT scan report in July 2021 suggestive of no new lesion in the chest wall (a, b). Post-radiotherapy changes and post-craniotomy status of the brain (c, d). New liver deposits are observed at multiple places in the liver (e, f).

**Figure 2:**
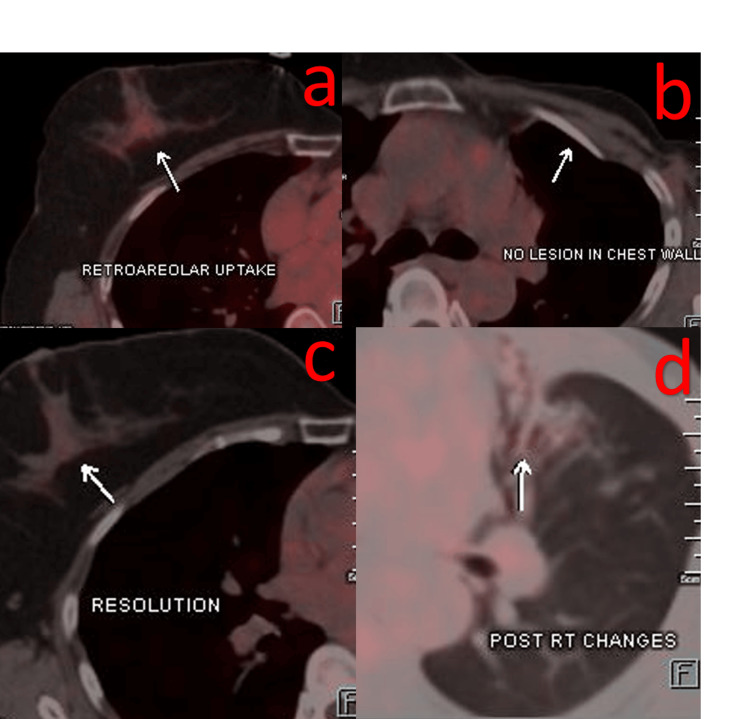
PET-CT scan report post 12 cycles of T-DM1 in March 2022 suggestive of resolution of previously seen retro areolar uptake in the right breast and no lesion in the chest wall (a, b). Resolution of liver deposits and post-RT changes (c, d).

A PET CT scan in July 2021 confirmed the absence of lesions in the left chest wall and regional nodes but identified new FDG-avid liver deposits in both lobes (Figure 1). Consequently, the patient’s therapy was shifted to T-DM1 (UJVIRATM) starting in July 2021, administered at a dose of 3.6 mg/kg every three weeks. Follow-up PET-CT scans after six cycles showed complete resolution of the liver deposits, although a suspicious, weakly metabolic nodular lesion was detected in the retroareolar region of the right breast (Figure 2).

**Figure 3:**
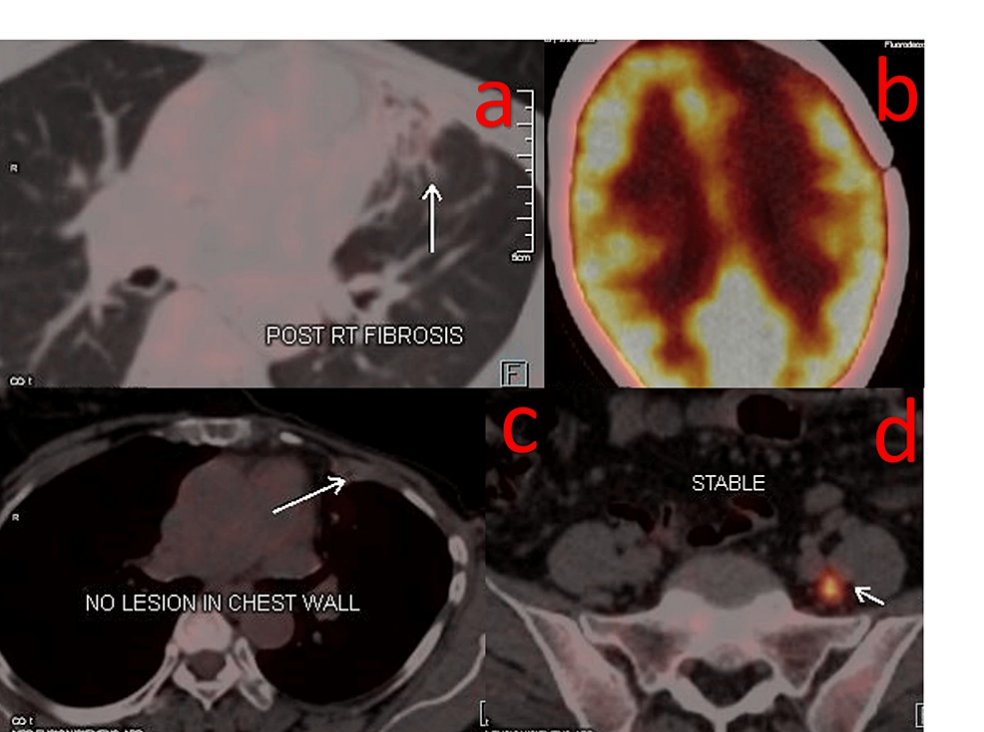
PET-CT scan report post 28 cycles of T-DM1 in February 2023 suggestive of stable disease and no lesion in the chest wall (c, d). Post-RT fibrosis and post-RT changes (a, b).

Subsequent PET-CT scans in March and August 2022 showed no signs of locoregional recurrence or distant organ involvement. However, a whole-body PET-CT in October 2022 revealed increased thyroid uptake suggestive of thyroiditis and a nodular lesion in the retroareolar region of the right breast. During this period, an MRI of the brain showed no evidence of disease recurrence. The patient continued on T-DM1 until April 2023, completing 31 cycles (Figure 3).

In April 2023, she developed a headache and vomiting. MRI reveals the most recent development of multiple ill-defined, heterogeneously enhancing brain lesions, with the largest measuring 34 x 2 mm in the left frontal lobe. These lesions were accompanied by significant surrounding edema, indicating metastatic involvement. Chronic small vessel ischemic changes were also noted in bilateral periventricular regions. Whole-brain re-radiation therapy was initiated in April 2023 to address these metastatic brain lesions. She was started on trastuzumab, capecitabine, tucatinib since then. Currently, she is on the same treatment and is clinically getting better. MRI done in August 2023 suggested a good response to brain lesions and no development of any newer lesions.

## Discussion

Brain metastases have become an increasingly prevalent challenge for individuals with HER2-positive advanced breast cancer. Individuals with human epidermal growth factor receptor 2 (HER2)-positive breast cancer are at an increased risk of developing metastatic disease in the central nervous system (CNS), especially in the brain [4].

The presence of brain metastases (BM) in HER2-positive metastatic breast cancer (MBC) is associated with unfavourable outcomes, and the available treatment options are constrained [5]. The limited therapeutic avenues underscore the complex nature of managing HER2-positive breast cancer when it extends to the brain, emphasizing the need for further research and the development of more effective interventions in this clinical context.

The landscape of systemic therapies for advanced HER2-positive breast cancer is rapidly changing. Currently, the preferred first-line treatment is a combination regimen of taxane, pertuzumab, and trastuzumab. For second-line therapy, the antibody-drug conjugate trastuzumab-emtansine (T-DM1) is the treatment of choice. For third-line and subsequent treatments, recommendations include lapatinib and/or trastuzumab-containing regimens and/or participation in clinical trials [6].

However, in the specific context of progressive HER2-positive breast cancer brain metastases, the treatment algorithm is less clear. Most efficacy data have been generated with lapatinib, a brain-permeable small molecule inhibitor of HER1/HER2, used either alone or in combination with other cytotoxic agents. Notably, response rates within the intracranial setting are generally less than 20%, and progression-free survival (PFS) is typically less than six months in the refractory setting [4]. This highlights the challenges and complexities associated with treating HER2-positive breast cancer when it metastasizes to the brain.

The conventional belief has been that larger monoclonal antibodies like pertuzumab and T-DM1 do not effectively cross the blood-brain barrier due to their size. However, recent evidence suggests that the blood-brain barrier undergoes changes in the presence of metastases, often referred to as the "blood-tumor" barrier. Radiolabeled studies of pertuzumab and T-DM1 have shown the accumulation of both molecules in brain metastases of HER2-positive breast cancer [4].

This revelation has important implications, indicating that systemic treatment involving HER2-targeted agents may enhance clinical outcomes in individuals with HER2-positive metastatic breast cancer (MBC) and brain metastases (BM). Survival for such patients has been reported to range from approximately 11 to 30 months [5]. This suggests that advancements in our understanding of the blood-tumor barrier and the ability of certain therapeutic agents to reach the brain may contribute to improved management and outcomes for individuals facing HER2-positive breast cancer with brain involvement.

Trastuzumab emtansine is an antibody-drug conjugate consisting of trastuzumab linked to emtansine, a microtubule inhibitor. This unique conjugate enables the intracellular delivery of emtansine to cells that overexpress HER2 via receptor-mediated endocytosis [3]. Following the proteolytic degradation of the antibody, emtansine is released and subsequently inhibits microtubule assembly. This mechanism leads to mitotic arrest and apoptosis.

Studies have demonstrated that trastuzumab emtansine (T-DM1) improves overall survival (OS) in patients with trastuzumab-resistant advanced metastatic breast cancer and asymptomatic brain metastases that were previously treated with radiotherapy. Notably, this improvement in OS was compared with the lapatinib plus capecitabine regimen. Additional small-scale studies have also indicated signs of clinical activity for T-DM1 in patients with HER2-positive MBC and BM [5]. These findings underscore the potential efficacy of T-DM1 as a treatment option for patients with HER2-positive breast cancer, especially those with brain metastases.

In a retrospective subset analysis of the EMILIA trial, 95 patients with asymptomatic baseline central nervous system (CNS) metastatic disease showed improved overall survival (OS) when treated with T-DM1 compared to lapatinib and capecitabine. Research has underscored T-DM1's ability to penetrate the modified blood-tumor barrier (BTB) [3].

Patients with a history of radiation treatment are likely to experience enhanced T-DM1 penetration. Multiple studies have indicated that whole-brain radiation therapy (WBRT) or stereotactic radiosurgery (SRS) can augment BTB permeability. Notably, recent small series involving HER2+ breast cancer brain metastases (BCBM) have demonstrated T-DM1's effectiveness, even in cases of treatment-resistant brain metastases. There are also case reports showcasing long-term treatment responses in patients with leptomeningeal disease who underwent radiation and T-DM1 therapy [3].

In this brief case report, we present a clinical scenario involving heavily pre-treated patients facing treatment-refractory Her2-positive brain metastases. These individuals experienced notable clinical benefits and radiographic response to T-DM1, with the patient maintaining therapy for more than 32 months. As of the current update, these patients not only remain alive but also exhibit measurable reductions in intracranial tumor size while being actively treated with tucatinib.

This data, when considered collectively, contributes to the growing body of evidence that underscores the clinical advantages of T-DM1 in the context of HER2-positive breast cancer with brain metastases. This underscores the importance of further exploration through appropriately powered early-phase clinical trials to delineate the treatment's benefits and assess its impact on the quality of life within this specific patient population.

## Conclusions

Brain metastases pose a significant clinical challenge for patients with advanced HER2-positive breast cancer. The ongoing development of new brain-permeable anti-HER2 treatments has steadily enhanced the effectiveness of systemic therapy for those with metastatic HER2-positive breast cancer.

The complexities of central nervous system biology, the distinctive local microenvironment, and the factually limited availability of clinical trials for patients with CNS involvement have collectively contributed to a less favourable prognosis for this group in the past. However, the introduction of a T-DM1 biosimilar in India now provides a more affordable option for patients who have exhausted standard treatments.
